# Experiences of teachers, educators, and school counselors about the sexual and reproductive health of educable intellectually disabled adolescent girls: a qualitative study

**DOI:** 10.1186/s12978-022-01397-8

**Published:** 2022-04-18

**Authors:** Shadi Goli, Farzaneh Rahimi, Marjan Goli

**Affiliations:** Nursing and Midwifery Sciences Development Research Center, Najafabad Branch, Islamic Azad University, Najafabad, Iran

**Keywords:** Intellectual disability, Iran, Teachers, Health educators, Counselors, Sexual health

## Abstract

**Background:**

Adolescents with intellectual disabilities are probably twice as many people without intellectual disabilities to be sexually abused by family members, caregivers, close relatives, and others in the community. Sex education and training are essential components of children's and teenagers' education and human rights, as well as a source of worry for parents and society. While the parents are thought to be the most accessible choice as sexual educators, they often do not fulfill this role. Therefore, professional teachers and trainers who have undergone sex education courses for mentally retarded adolescents are more reliable sources to provide the sexual information in terms of their educational role. This study aimed to determine the experiences of teachers, educators, and school counselor parents regarding the sexual and reproductive health of educable intellectually disabled adolescent girls.

**Methods:**

This was a qualitative content analysis study. 35 participants were selected via purposive sampling with maximum variation, and data were collected through in-depth individual interviews, focus group discussions and field notes, and analyzed using the conventional qualitative content analysis method simultaneously.

**Results:**

Three subcategories have emerged: “knowledge and professional experience of teachers, educators, and school counselors with how to educate and care for adolescent sexual health”, “proficiency of teachers, educators, and school counselors in guiding families in solving their child's sexual problems”, “attitude of teachers, educators, and school counselors towards sexual behaviors and sexual education of adolescents” which formed the main category of “teachers, educators, and school counselors’ inefficiency in maintaining ID adolescent girls’ sexual and reproductive health”.

**Conclusions:**

Teachers, educators, and school counselors encounter a variety of issues related to the sexual and reproductive health of intellectually impaired teenage females, as a consequence of the findings. As a result, efforts should be made to enhance knowledge and skill development, as well as the evolution of negative attitudes. Therefore, the teaching of sexual guidelines for teenagers with mental impairments should be included in the agenda of the country's educational policies. Teachers and educators should be taught by health experts via the holding of in-service training courses.

## Plain English summary

Achieving sexual health is a key task to develop health for all people including adolescents. Adolescents with intellectual disabilities are probably twice as many as people without intellectual disabilities to be sexually abused by family members, caregivers, close relatives, and others in the community. The role of schools and educators to manage children's sexual behaviors and provide appropriate training in this field was accepted by most societies. This research aimed to ascertain instructors', educators', and school counselors' perceptions on teenage females with educable ID's sexual health. Participants in the present study were mothers, teachers, educators, and school counselors who were selected via purposive sampling in Isfahan from July 2017 to April 2018. Data were collected from via semi‑structured interviews, focus group discussions, and field notes, and analyzed using conventional content analysis. The results showed that teachers, educators, and school counselors face different challenges about the sexual and reproductive health of intellectually disabled adolescent girls. Therefore, it should be attempted to promote knowledge and skill and eliminate negative attitudes and make them aware of the sexual rights of intellectually disabled adolescent girls to protect themselves in different situations.

## Introduction

Achieving sexual health is a key task to develop health for all people, including adolescents [1]. Sexual health is an individual's ability to express his or her sexuality within the framework of society's value system, laws, beliefs, and current culture without fear of sexually transmitted infections, unwanted pregnancies, coercion, violence, and discrimination [2]. Both people with disabilities and people without disabilities exhibit similar sexual development characteristics. According to World Association for Sexual Health (2014), sexual rights form the basis of international human rights. As a result, democratic education opportunities must be established to demonstrate that each person is unique, equal, and must be self-sufficient, regardless of whether they have or do not have disabilities [3]. But, in most societies, sexuality, which is considered a form of pleasure for everyone, is still ignored as part of the rights of people with intellectual disabilities [4]. Regarding intellectual limitations, adolescents with intellectual disabilities are deprived of educational resources that may require intellectual ability to use them, and on the other hand, their limited relationship with society intensifies this process [5]. Adolescents with intellectual disabilities are probably twice as many as people without intellectual disabilities to be sexually abused by family members, caregivers, close relatives, and others in the community [6, 7]. Sexual abuse can have long-term consequences such as incompatibility, poor sexual function, high-risk sexual behaviors, unwanted pregnancies and the risk of STIs and HIV / AIDS; repeated sexual abuse, and eventually incidence of anxiety and depression in adolescents [8].

According to the United Nation (UN)'s Sustainable Development Goal (e.g. Leave no one behind), equal access to sex education for all people, including the disabled and vulnerable, is a human right [9]. However, the sex education of children and adolescents, as well as a concern of parents and society; nonetheless, children and adolescents continue to be denied this form of education globally [1].

Whereas teachers and parents play an important role to prepare the adolescents with intellectual disabilities to develop healthy relationships and protect themselves in high-risk situations [10]. Sex education can lead to positive self-image, self-confidence, gender equality, and prevention of sexual abuse in adolescents with intellectual disabilities [11]. Supporting people with disabilities via sex training can improve their quality of life [12].

Despite the research evidence that people with intellectual disabilities engage in sexual activities, there are misconceptions about their sexuality that prevent them from accessing proper sex training and social acceptance [13]. Typically, parents bear the primary duty for sex teaching in persons with intellectual impairments. However, there are obstacles such as parents' fear of instilling ideas in adolescents that may result in sexual behavior, denial of their child's evolving sexual desires (sexuality), and a lack of knowledge and skill in effectively communicating this information that parents face in their role as sexual educators. Thus, while parents are thought to be the most accessible as sexual educators, they often do not fulfill this role [14].

In Iran, regarding the traditional structure of families, talking about sexuality is taboo, and raising sexual issues with adolescents, especially girls, by parents, caregivers and teachers is regarded shameful. Adolescents 'ignorance; parents' ignorance and wrong attitude on sexual issues have caused parents to hide or deny their adolescent sexual problems, especially cases of sexual harassment, to protect the family's reputation. Uninformed parents mistakenly believe that adolescents with mental retardation are different from adolescents with normal Intelligence Quotient score [13]. Parents, especially fathers, despite their poor knowledge about adolescent sexual behaviors and problems, do not have the desire to receive education and information about how to properly deal with adolescent sexual inappropriate behaviors, and therefore when faced with these behaviors are concerned [13]. Not only are parents reluctant to receive sex education, but they also have a negative attitude toward their adolescents' sex education. According to the findings, parents cannot be knowledgeable and motivated educators for mentally retarded adolescents in the field of sex, and therefore it seems that the education organization has a key role in educating parents and adolescents with mental disabilities in the field of sexuality [15]. Therefore, professional teachers and trainers who have undergone sex education courses for mentally retarded adolescents are more reliable sources for providing sexual information due to their educational role [14].

The role of schools and educators to manage the children's sexual behaviors and provide appropriate training in this field was accepted by most societies [16, 17]. Reviewing and developing sex training at schools of some communities is not an easy matter, and there is still fear and anxiety about talking about sexual issues [18]. One of the effective factors in school-based sexual education is the ability of teachers who are responsible for this education. Besides, it was argued that teachers' attitude is also very influential in the effectiveness of this training. In this field, researchers recommend that before teachers participate in the presentation of sexual education programs, their attitude and trust toward sexual skills training should be evaluated. Research showed that most teachers do not feel ready to manage and teach adolescent sexual issues, and in most cases, consider sexuality only from a biological point of view and not in a social context. Because adolescents regard teachers as the most reliable source of information on sexual issues [19], and because no special study has been conducted on the challenges of teachers, educators, and school counselors in the field of sexual health of intellectually disabled adolescent in Iran, the need for examination of these issues becomes apparent.

## Methods

### Study design

The present qualitative research used a content analysis approach which was conducted from July 2017 to April 2018.

### Settings, sample and recruitment

Participants included 8 mothers, 15 teachers, 8 educators, and 4 school counselors who were living in Isfahan, Iran and were selected via the purposive sampling.

Then, sampling was continued with maximum variation (in terms of age, educational level, occupation and socioeconomic status). Participants were accessed via visitation to the Behzisti centers and schools for the adolescent with special educational needs.

The inclusion criteria included mothers who had an educable ID adolescent girl within the age range from 11 to 20 years, lack of any diagnosed psychological disorders, willingness to participate in the study, and giving informed consent. At least five years of experience working as teachers, educators, and school counselors was required for inclusion. Once contacted, none of the eligible volunteers declined to participate in the research.

### Data collection

Data were collected via semi-structured in-depth face-to-face interviews, Focus Group Discussions, and field notes. The first author (SG), who has 12 years working experience in reproductive and sexual health and was Ph.D. candidate in reproductive health., conducted the interviews and field notes. Other authors have previous interviewing experience and qualitative paper/report writing. Prior to data collection, the first author wrote down initial preconceptions and beliefs about the research topic based on her previous working experience and from a review of the literature. Interview and Focus Group Discussions began with open questions such as “what problems have you encountered with your adolescent about sexual issues?” (For mothers) or “based on your experiences, what are the problems of ID adolescent girls regarding their sexual health?” (For other participants) and continued with probing questions.

In this study, 45–100 min interviews were conducted in locations chosen by the participants. Interviews continued until reaching data saturation.

### Data analysis

Data analysis was carried out using the conventional qualitative content analysis [20]. Any software was used to analyze the data. The first author (SG) transcribed the data on a regular basis as each interview was conducted and recorded using an mp4 player. The interviews were then re-examined to ensure that they were completely understood. After coding the sentences and phrases inductively, the identical codes were merged, and those with a similar notion were grouped together and organized into sub-categories. Subsequently, using the inductive technique, the sub-categories were compared, and related sub-categories were combined into a single major category [21].

### Rigor and trustworthiness

To ensure the trustworthiness of obtained content, coded interviews were consulted with four participants in other sessions, and their final comments were summarized to allow participants to review the interviews. To ensure the findings' credibility, several methods were used, including in-depth interviews at various times and locations, combining several data collection methods, such as individual interviews and field notes, and randomly selecting participants from the various groups (mothers, teachers, educators, and school counselors) with the greatest variation. To enhance transferability, the findings of the research were shown to five persons (3 teachers and 2 educators) with comparable qualities to the participants who did not participate in the study, who were asked to rate how similar the study's results were to their own experiences. The views of four experts were also utilized to check that the results were compatible with the participants' remarks.

### Ethical considerations

The research approval of the Institutional Ethics Committee of Islamic Azad University branch of Najafabad (ethical code: IR.IAU.NAJAFABAD.REC.1397.003) was obtained, and informed consent, anonymity, the confidentiality of information, and right of withdrawal at any time were considered.

## Results

Tables 1 present the demographic characteristics of participants. During data analysis, 40codes and three subcategories were developed. Three subcategories under the headings: “Knowledge and professional experience of teachers, educators, and school counselors with how to educate and care for adolescent sexual health,” “Proficiency of teachers, educators, and school counselors in guiding families in solving their child's sexual problems,” “attitude of teachers, educators, and school counselors towards sexual behaviors and sexual education of adolescents”wereemerged which formed the main category under the heading: “teachers, educators, and school counselors’ inefficiency in maintaining ID adolescent girls’sexual and reproductive health” (Table 2).

**Table 1:** Demographic characteristics of participants (teachers, educators, school counselors and mothers)

Age	26–48 years
Gender	Female (35)
Marital status	Married (28), Single (7)
Educationallevel	Elementary school (5), Diploma (3), Bachelor’sdegree (27)
Job	Housewife (6), Employee (2), Teacher (15), Educator (8), School counselor (4)
Workingexperience	3–25 years

**Table 2:** Results of data analysis

Codes	Sub‑category	Main category
Lack of skills of the educators, teachers and school counselors in adolescent sexual health care Lack of education sexual health issues to adolescents by teachers, educators, and school counselors Lack of the knowledge and skills of educators, teachers andschool counselors in education sexual issues to ID adolescent girls Lack of sex education courses for ID adolescent girls Lack of retraining courses for teachers, educators, and school counselors	Knowledge and professional experience of teachers, educators, and school counselors with how to educate and care for adolescent sexual health	Teachers, educators, and school counselors’ inefficiency in maintaining ID adolescent girls’ sexual and reproductive health
Lack of information of teachers, educators, and school counselors Failure to provide education related to adolescent sexual issues to parents in Behzisti centers and schools Lack of knowledge and skills of teachers, educators, and school counselors in holding effective sex education sessions for parents Lack of clear education of mothers due to the shame of revelation of teachers, educators, and school counselors	Proficiency of teachers, educators, and school counselors in guiding families in solving their child's sexual problems	
Lack of education of mothers due to negative attitude of teachers Lack of guidance from mothers due to fear of getting involved in legal issues Lack of education of mothers by teachers, educators, and school counselors due to the taboo of sexual issues	Attitude of teachers, educators, and school counselors towards sexual behaviors and sexual education of adolescents	

### Knowledge and professional experience of teachers, educators, and school counselors with how to educate and care for adolescent sexual health

The data analysis revealed that teachers, educators, and school counselors in welfare and exceptional education centers have a limited understanding of sexual problems among adolescents with intellectual disabilities, as well as how to care for and protect them from sexual harassment and abuse; they also lack the knowledge necessary to educate adolescents with intellectual disabilities about maintaining their health. Teachers, educators, and school counselors stated that most of them have acquired information related to sexual issues in the adolescents with intellectual disabilities based on experience and has not received comprehensive formal training on the sexuality of adolescents with intellectual disabilities and how to properly deal with their sexual behaviors.

*"Since educators work with children, they unwantedly get a series of information about this case, but it would certainly be much better if training classes held on the sexual problems of adolescents with intellectual disabilities." Educators usually obtain their information either through experience or ask questions from advisors. "(Educator).*

*"The Information level of teachers and assistants of schools about the sexual issues of adolescents with intellectual disabilities is very low. It's to the same extent of in-service courses that hold us about sexual issues which these courses deal with just a few basic issues like menstruation and menstrual hygiene, and raise the sexual issues of adolescents without intellectual disabilities, not a class for adolescents with intellectual disabilities"(Teacher).*

Teachers, educators, and school counselors stated that one of the most common sexual behaviors in adolescents is masturbation, which occurs in public places, including schools. Moreover, genitals view and showing private parts of the body to others is seen in these adolescents that teachers, educators, and school counselors do not have enough knowledge to properly deal with these sexual behaviors in adolescents and control and educate them.

*"They have a lot of masturbation; of course, it is more at home, but we also see it at school, and whatever we warn them, it is useless. Sometimes we even have to punish them, because it is bad for other children as well, but it is useless. We really do not know how to deal with these behaviors of adolescents. Unfortunately, the educators—even though they studied psychology- do not behave properly with the child in these situations. I wish they would hold us a training class and talk about these issues. This year, we had a case where a girl took her dress off at class, showed her bra to the others, opened her bra, and showed her breasts to her friends. At these times, I am really do not know how to behave." (Teacher).*

### Proficiency of teachers, educators, and school counselors in guiding families in solving their child's sexual problems

According to the mothers who participated, teachers do not express sexual issues and subjects in adolescents explicitly and plainly during parent training sessions held at schools, because parents are portrayed as sexually mixed (fathers attend the sessions) and raising sexual issues is taboo. As a result, parents do not receive adequate and proper training to maintain their adolescent's sexual health. *rape and these things, but it is not clear. "Because both moms and dads are present at these sessions and they do not explain clearly very much because of the men, and we do not really understand how we should solve our adolescent's problems."(Mother).*

Participating mothers stated that teachers, educators, and school counselors, even in private counseling sessions, do not provide useful and comprehensive guidance on how parents deal with adolescent sexual behaviors such as self-stimulation because of their shame and modesty or low level of information.

*"Every time I refer to school, even when I talk privately with the school counselor, finally I do not realize how I should solve my problem. I do not know it is due to shame and modesty that the consultant does not explain openly and clearly to me or that she herself does not really know what to do in such situations. When I ask a lot of questions, she says I don't have time anymore and introduces me some books. I really do not know how to solve this problem of my daughter's excessive masturbation. She always sleeps as groveled and do this work. Whatever I distract him, it is useless again. I'm very sad."(Mother).*

### Attitude of teachers, educators, and school counselors towards sexual behaviors and sexual education of adolescents

A number of the teachers, educators, and school counselors believed that these adolescents had more sexuality than adolescents without intellectual disabilities and believed that sex training to these adolescents would cause to stimulate the adolescent's sexuality and motivation and do high-risk sexual behaviors.

*"I think these girls have high sexuality. They cling to other girls, kiss them. They even hug us. From their looking, the way they talk, it has appeared that they have more sexuality than people without intellectual disabilities."(Educator).*

*"I think they will be stimulated if we teach sexual training to these children. Because they always want to get married, it will get worse if we explain to them anymore."(Teacher).*

Some mothers, teachers, educators, and school counselors stated that sexual issues training would eliminate shame, modesty, and taboo of these issues among adolescents by creating curiosities in adolescents.

*"If we involve teenage girls to this training, it may create curiosities in her, and she herself may cause to disappear taboo of this work." (Counselor)*

Additionally, teachers and school counselors claimed that they were hesitant to provide sex education for teenagers because of fear of being accused of teaching immoral topics to children by school authorities or parents, as well as a lack of support from education officials and legal implications. *"One issue that we are concerned about is that we ourselves will be accused of saying that they are teaching sexual relations or contraception ways to unmarried girls, which means that it does not matter if you want to do this work. We are really afraid of the consequences of these trainings and legal problems and parents' complaints and reprimand by the school principal. "In fact, no one feels ownership over this matter."*

*(Teacher)*

Some teachers, educators, and school counselors also stated that sex training is considered a socio-cultural taboo.

*"Sexual issue is taboo in our culture. We cannot talk about taboos. It is better to talk about sexual issues in the privacy of the family without going into details."(counselor)*

## Discussion

The present study was conducted to determine the experiences of teachers, educators, and school counselors regarding the sexual health of educable ID adolescent girls. According to the results, teachers, educators, and school counselors of welfare and Behzisti centers and schools for the adolescent with special educational needs are not sufficiently aware of the sexuality and sexual behaviors of adolescents with intellectual disabilities and have incorrect attitudes in this regard. They are unable of educating and caring for these teenagers' sexual health, as well as assisting families in resolving their child's sexual difficulties, due to a lack of ability. Participants believed that teachers, educators, and school counselors are unfamiliar with how to educate and care for the sexual health of adolescents. Participating teachers, educators, and school counselors stated that in most cases, they obtained sexual information about adolescents with intellectual disabilities based on experience and did not receive comprehensive formal training on the sexuality of adolescents with intellectual disabilities and how to properly deal with their sexual behaviors. In this regard, a research conducted in Tanzania discovered that the majority of teachers had fundamental understanding about sex and sexuality, which they obtain from personal studies (i.e., reading), experience, and the media. Teachers said that there were no sex education programmes in which they could be trained about sex education [22].

The participants believed the most common sexual behaviors in the adolescents with intellectual disabilities, which takes place in public places, including the school, are masturbation, showing genitals organ, and showing private parts of the body to others that teachers, educators, and school counselors do not have enough skills and knowledge to properly deal with these sexual behaviors of adolescent and control and educate adolescents. Other studies have shown that many instructors lack the essential preparation and self-confidence to deal with sexual problems or sexual training of adolescents with intellectual impairments and overlook the advantages of sexual training in preventing sexual harassment and sexual abuse of adolescents [23–25]. Abolghasemi et al. found that educators of schools do not have enough knowledge to deal properly with students 'sexual behaviors and consider some of the students' normal behaviors as abnormal and when some sexual behaviors occur in students, they show behaviors such as opposition, interrogation, and reprimand. Therefore, a child with normal sexual behavior may be introduced as a problematic child at school and they believed that concealing sexual issues, blaming and interrogation of the student and induction of anxiety in them are not appropriate functions. It seems that the lack of guidance and instructions for dealing with children's sexual behaviors exposes these educators to judging and drawing conclusions about behaviors based on their attitudes and the structure of their sexual education [18].

Although the best and most accessible counseling reference for the adolescents is school counselors, these counselors usually only provide academic counseling and lack the necessary skill in counseling and sexual education which have not received any training in this field [26]. There is usually no single sexual training policy in schools and Behzisti centers, and most trainings are based on individual choice; as a result, there is no comprehensive program and appropriate to adolescent development [27]. In societies such as Iran, where sex training is neither included in students' educational curriculum nor in training programs of teachers, educators, and school counselors, their insufficient knowledge and skill are not surprising and far from expected. It is obvious that teachers, educators, and school counselors always suffer from the lack of specialized and academic training in the field of sexual issues. Also, without regard to the specific content and training packages, teachers, educators, and school counselors are unable to answer students' sexual questions.

Based on this study's results, the educational content taught to adolescents by teachers, educators, and school counselors in most cases are teaching topics such as puberty, menstruation, personal hygiene; and important and sensitive topics such as intimate relationships, finding friends, marriage, privacy-preserving and common sexual behaviors such as self-stimulation, showing genitals organ and sexually transmitted diseases such as AIDS are not taught for some reasons such as the ignorance of teachers, educators, and school counselors, the presence of shame and modesty, being taboo of sexual topics and their misconceptions. In this regard, the researchers in other studies mentioned that adolescents with intellectual disabilities receive limited sex training in schools, and the training content taught does not cover important topics such as HIV/AIDS[28, 29].

According to Girgin-buyukbayraktar et al., teachers, family members, caregivers, and special education specialists who provide self-care for individuals with intellectual disabilities encounter issues with sexual and intimate relationships involving incorrect societal stereotypes, handicaps in obtaining information, overprotective parenting and a lack of places to meet, inability to control sexual urges, and being unable to control sexual urges [30]. The results of a study in Kenya revealed that teachers emphasized the need for sex training for adolescents with intellectual disabilities, and teaching basic topics such as sexual relation, intimacy, pregnancy, condom-use and other methods of contraception, prevention of sexual abuse was considered necessary to empower these adolescents [28]. Saxe and Flanagan found that teachers and educators should be able to express training content in accordance with the understanding of adolescents with intellectual disabilities and via appropriate and diverse training methods such as using the images, paintings, photographs, videos, etc. for the adolescent. On the other hand, collaboration between teachers and educators, as well as officials and administrators, to develop programs that educate people about persons with intellectual impairments and their human and sexual rights, may help these teenagers maintain their sexual health. It is necessary to design sexual guidelines special for training the adolescents with intellectual disabilities and to teach these guidelines to educators and teachers periodically and continuously. The existence of a specific guideline for sexual education of adolescents with intellectual disabilities helps educators and teachers overcome training barriers such as personal moral judgments, emotional vulnerability, lack of confidence in dealing with adolescent sexual issues, and a lack of skill to manage the situation appropriately [31].

According to the findings of this research, instructors, educators, and school counselors perform poorly in assisting parents to resolve their child's sexual issues. Because of the mixed nature of the education sessions and the presence of fathers in the sessions, mothers believe that teachers, educators, and school counselors do not express the sexual issues of adolescents explicitly and clearly; as a result, parents do not receive appropriate and adequate training to maintain their adolescent's sexual health. Mothers stated that teachers and educators, even in private counseling sessions, do not provide helpful and comprehensive guidance on how to deal with sexual behaviors of adolescents such as self-stimulation in terms of their shame and modesty or low level of information and the sexual issues of adolescents with intellectual disabilities have caused anxiety and concern of parents. As a result, it seems that separate educational sessions for mothers and dads of adolescents with intellectual impairments should be held by professional instructors, educators, and school counselors so that parents may discuss their child's sexual difficulties without feeling embarrassed. According to Abolghasemi et al. parents do not have enough confidence to the skill of teachers in guiding their children. Therefore, there is no necessary interaction between home and school. While these instructors seem to be confident in their skills, they view sexual education in schools as a matter of personal privacy. Due to the school's lack of a clear executive policy and the hostility of parents (particularly mothers) to holding sessions on sexual training, parents and school are unable to share learning experiences, resulting in both groups lacking appropriate competence in dealing with sexual problems [18]. UNESCO (2009) emphasizes the key role of trained teachers in sexual training. It seems that if schools perform well and provide high-quality sexual training programs for parents, they will be a good place to teach responsible decision-making skills in sexual life and answer the ambiguities of parents to solve their children's sexual problems and by empowering the parents and providing motivation, enable them to guide their adolescent sexually and control risky sexual behaviors [32]. In this regard, researchers in other studies mentioned that in order to solve sexual problems, individuals with intellectual disabilities, their teachers, and parent should cooperate [30, 33]. Therefore, the optimal interaction between school and home is considered one of the most fundamental issues in the country's education system which can perhaps be mentioned as a missing link of education and training.

Some of the teachers, school counselors, and educators participating in the present study had misconceptions and negative attitudes about the sexuality of adolescents with intellectual disabilities. They felt that these teenagers had greater sexuality than adolescents without intellectual impairments and that sexual education stimulated their libido and increased the prevalence of high-risk sexual practices. Teachers, educators, and school counselors expressed false views, such as that teenagers with intellectual impairments are "hypersexual" and unable to regulate their sexuality. They are afraid that after sexual education, these teenagers would want to experience what they have learnt, putting them at risk for unhealthy relationships, unexpected pregnancies, or sexually transmitted illnesses. In this regard, researchers in other studies mentioned that teachers and educators usually avoid adolescent's training for fear that adolescents may show the inappropriate sexual behaviors after sexual training, and consequently, due to lack of knowledge, the adolescent is unable to establish proper relationships with others [24, 31, 34]. According to Jalali Aria et al., from the perspective of teachers and parents, the best time to teach most sexual issues is marriage time, and most of them were opposed to training before marriage and adolescence [35]. In contrast, Haglund found that educators, parents, and physicians believed that early sexual education protected adolescents from premature sexual activity and unwanted pregnancies. On the other hand, they believed that early and continuous education are a quick way to deal with and neutralize inaccurate information [36]. Hosseini Khanzadeh et al. found that 92% of teachers and 83% of parents emphasized the role of sexual training in protecting people with intellectual disabilities from sexual exploitation [37].

Teachers, educators, and school counselors' restrictive attitude that discussing sexual issues with adolescents with intellectual disabilities is unnecessary and risky may eliminate the taboo surrounding sexual issues in adolescents, limit their performance, and decrease their motivation to acquire skills. In other studies, researchers found that some instructors and educators oppose sexual education for teenagers with intellectual impairments because it may cause them to become more irresponsible and uncontrolled [29, 38]. Based on this study's results, teachers, educators, and school counselors often prefer not to deal with sensitive issues such as sexual issues, because they are worried about being criticized by school officials, parents or the community, which is in line with the findings of Pokharel’s study [39]. In the present study, some participants have a negative attitude towards the sexual rights of adolescents with intellectual disabilities due to their religious beliefs and cultural beliefs, which was also mentioned in the study of Aderemi [40].

As a result, sexual health practitioners should work to dispel these false ideas and taboos by fostering a culture of understanding among instructors, educators, and school counselors. They should be trained how to provide accurate, scientific, nonjudgmental information that is age and development appropriate, as well as how to maintain adolescent sexual health via a systematic and rigorous school-based teaching method. Without this instruction, the teenager will be exposed to incorrect and dangerous knowledge via peers, media, and other sources. Sexual training should be in accordance with the culture and values of society. Sexual training includes a range of relationships, not just sexual relations. Well-trained teachers, educators, and school counselors can teach adolescents about sexual issues openly and responsibly to deal with the effects of content provided by electronic media, which are not controlled.

## Conclusion

According to the findings, instructors, educators, and school counselors lack the essential training and experience to educate and handle sexual problems among teenagers who have intellectual impairments and encounter a variety of obstacles. Since teachers, educators, and school counselors are the most reliable and trustworthy sources of information for adolescents and can have a positive impact on adolescent sexual life, so it should be attempted to promote knowledge and skill and evolve negative attitudes and make them aware of the sexual rights of intellectually disabled adolescent girls to protect themselves in different situations. Therefore, the education of sexual guidelines special for educable intellectually disabled adolescent girls by health professionals through the holding of compulsory in-service training courses should be included in the agenda of educational policies.

## Data Availability

The datasets generated and/or analyzed during the current research are notpublicly available as individual privacy could be compromised but are available from the corresponding author on reasonable request.

## Abbreviations

ID: Intellectually disabled
STIs: Sexually transmitted infections
HIV: Human immunodeficiency virus
AIDS: Acquired immune deficiency syndrome
